# Inelastic collisions of ultracold triplet Rb_2_ molecules in the rovibrational ground state

**DOI:** 10.1038/ncomms14854

**Published:** 2017-03-23

**Authors:** Björn Drews, Markus Deiß, Krzysztof Jachymski, Zbigniew Idziaszek, Johannes Hecker Denschlag

**Affiliations:** 1 Institut für Quantenmaterie and Center for Integrated Quantum Science and Technology IQ^ST^, Universität Ulm, D-89069 Ulm, Germany; 2 Faculty of Physics, University of Warsaw, Pasteura 5, 02-093 Warsaw, Poland

## Abstract

Exploring and controlling inelastic and reactive collisions on the quantum level is a main goal of the developing field of ultracold chemistry. For this, the preparation of precisely defined initial atomic and molecular states in tailored environments is necessary. Here we present experimental studies of inelastic collisions of metastable ultracold Rb_2_ molecules in an array of quasi-1D potential tubes. In particular, we investigate collisions of molecules in the absolute lowest triplet energy level where any inelastic process requires a change of the electronic state. Remarkably, we find similar decay rates as for collisions between rotationally or vibrationally excited triplet molecules where other decay paths are also available. The decay rates are close to the ones for universal reactions but vary considerably when confinement and collision energy are changed. This might be exploited to control the collisional properties of molecules.

## Abstract (web summary)

Investigating the collisional behaviour of molecules on the quantum level is the key in understanding and controlling chemical reactions. Here the authors measure inelastic collision rates for ultracold Rb_2_ dimers in precisely defined quantum states and show that the rates can be tuned via external parameters.

Recent advances in the preparation of ultracold molecular samples in well-defined quantum states^1^,^2^,^3^,^4^,^5^,^6^,^7^ sparked increasing interest in studying molecular collisions and chemical reactions on a pure and fundamental level^8^,^9^,^10^,^11^,^12^. Such experiments were first carried out with highly excited molecules^13^,^14^,^15^,^16^,^17^,^18^,^19^,^20^,^21^,^22^,^23^,^24^ (also in the context of Efimov physics^25^,^26^,^27^,^28^,^29^,^30^), which can vibrationally relax in a collision. For molecules in the vibrational ground state this decay is barred, but other interesting reaction paths remain. First investigations of reactive or inelastic loss of singlet molecules in the rovibronic ground state with polar KRb and RbCs have recently been carried out^5^,^31^,^32^,^33^,^34^. Besides the electronic ground state 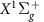, the metastable triplet state 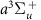 is of special interest for collision experiments. If collisionally long-lived, such triplet molecules would allow for many interesting applications, such as tunable Feshbach resonances, due to their sizeable magnetic moment. To investigate chemical reactions between cold molecules, optical lattices are a convenient testbed as they allow for either isolating the molecules from each other or letting them collide. In addition optical lattices offer the possibility to control the dimensionality of the scattering process and to tune the interaction^35^,^36^,^37^,^38^. On the basis of this approach, it has been shown that strong inelastic collisions induce correlations and can inhibit particle loss in a molecular sample, a manifestation of the quantum Zeno effect^15^,^34^.

In this work, we present measurements on ultracold collisions of metastable triplet molecules that are internally in their lowest energy state. Specifically, we use ^87^Rb_2_ dimers of the 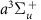 state which are in the lowest hyperfine level of the rovibrational ground state. As reference measurements, we carry out collision experiments with rotationally excited molecules (rotational quantum number *R*=2) and with vibrationally highly excited Feshbach molecules. Initially, the dimers are prepared in a cubic 3D optical lattice with at most a single molecule per lattice site (see Methods). By quickly ramping down one of its directions, the lattice is converted into an array of quasi-1D potential tubes (Fig. 1a). Subsequently, molecules within the same tube collide with tunable relative energies on the order of μK × *k*_B_, far above the Tonks gas regime of the work of Syassen *et al*.^15^. A single tube is typically filled with only a few molecules and can be considered as a closed few-body system since tunnelling between the tubes is negligible.

Whenever a collision between two molecules is inelastic or reactive, enough energy is released to expel all products out of the lattice. This is because the lattice depths are comparatively shallow, being on the order of about 10 μK × *k*_B_, which corresponds to ∼210 kHz × *h*. After a given interaction time *t* we measure the total number of remaining Rb_2_ molecules *N*(*t*) and the width of the whole cloud 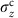
(*t*) along the tubes. From the observed decay of *N* we conclude that a large part of the molecules is already lost in the first possible collision, quite independently of the internal vibrational excitation. Using a simple model we extract the decay rate constants and investigate how they depend on the confinement of the potential tubes. These results are then compared to predictions of a quantum defect model^39^,^40^.

## Results

### Collisions of Feshbach molecules

In the following, we first discuss the collision experiments with Rb_2_ Feshbach molecules as this will help us analysing the data for the *v*=0 states. Figure 2a shows three data sets of *N*(*t*) corresponding to different confinements of the potential tubes. We observe a strong loss of molecules within the first tens of milliseconds. It is striking that the decay takes place in a step-wise fashion which, after ∼100 ms, gives way to a much slower exponential decay with a corresponding time constant of >1 s. This slow exponential decay is similar to the one we observe in a deep 3D optical lattice (Fig. 2a, inset), which is due to background gas collisions and spontaneous photon scattering^41^. Figure 2b shows that the width 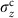 oscillates synchronously to the steps.

We interpret these dynamics as follows: as one direction of the 3D lattice is quickly ramped down (within 400 μs), the particles are suddenly released from their individual lattice sites into one-dimensional (1D) tubes. Along these tubes there is a harmonic confinement with trap frequency *ω*_*z*_ due to the Gaussian intensity profile of the 2D lattice laser beams. The molecules will synchronously undergo an oscillatory motion along this direction with a period *T*=2*π*/*ω*_*z*_, while being strongly confined transversally. As a result, the width of the observed cloud 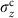 oscillates with 2*ω*_*z*_ (Fig. 2b). Whenever the cloud is small and dense, the probability for molecular encounters and losses is increased. On the other hand, if the cloud is large the dimers are separated from each other and *N*(*t*) stays almost constant. Thus, the longitudinal oscillatory motion explains the step-like decay of the molecules. Once all inelastic collisions have taken place the fast losses stop and the remaining signal corresponds to single molecules in the tubes. This regime is typically reached after 50–100 ms.

### Modelling of collision dynamics and analysis

**Figure 1:**
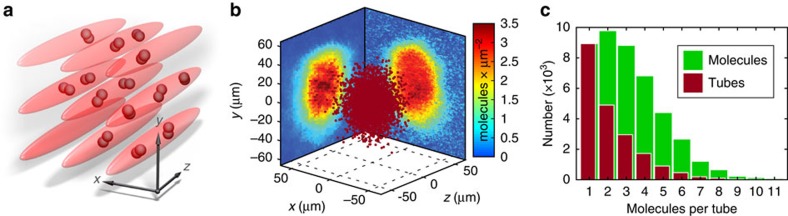
Initial spatial distribution of molecules. (**a**) Illustration of molecules confined in an array of quasi-1D traps (potential tubes) within which they can collide. (**b**) Random-generated Gaussian-shaped molecular cloud (red dots) of 4.3 × 10^4^ Feshbach molecules which matches the observed molecular distribution as determined by the absorption images in the *y*, *z*- and the *x*,*y*-plane. (**c**) Histogram of the molecular occupation of tubes as inferred from **b**. The red bars count the number of tubes with a given filling, while the green bars count the total number of molecules located in these tubes.

To model the molecular decay in a quantitative way, we first reconstruct the distribution of the molecules in our 3D optical lattice. The molecules are initially produced from a Gaussian-shaped cloud of ultracold atoms in the optical lattice via magnetic Feshbach ramping (see Methods). We assume Poisson statistics for the atomic occupation of each individual lattice site. Only lattice sites that are occupied by exactly two atoms will finally be occupied by a single molecule. All other lattice sites will end up empty. Thus, a given total atom number and cloud size fixes the initial molecular distribution, see Fig. 1b. We have verified that the predicted cloud size and total number of the Feshbach molecules indeed agree with our measurements (see Methods). Taking the molecule distribution of Fig. 1b the histogram of the resulting filling of the 1D tubes is depicted in Fig. 1c. The average occupation is ∼2.2. The histogram helps us to gain additional insights. If we assume inelastic two-body collisions to be the only source of particle loss, then the number of molecules remaining after a decay time of ≈100 ms equals the number of tubes with an odd initial filling. Evenly occupied ones, by contrast, end up empty. By comparing the experimentally measured fraction of remaining particles to this prediction we can check the model for consistency (see Supplementary Note 1 and associated Supplementary Fig. 1).

**Figure 2:**
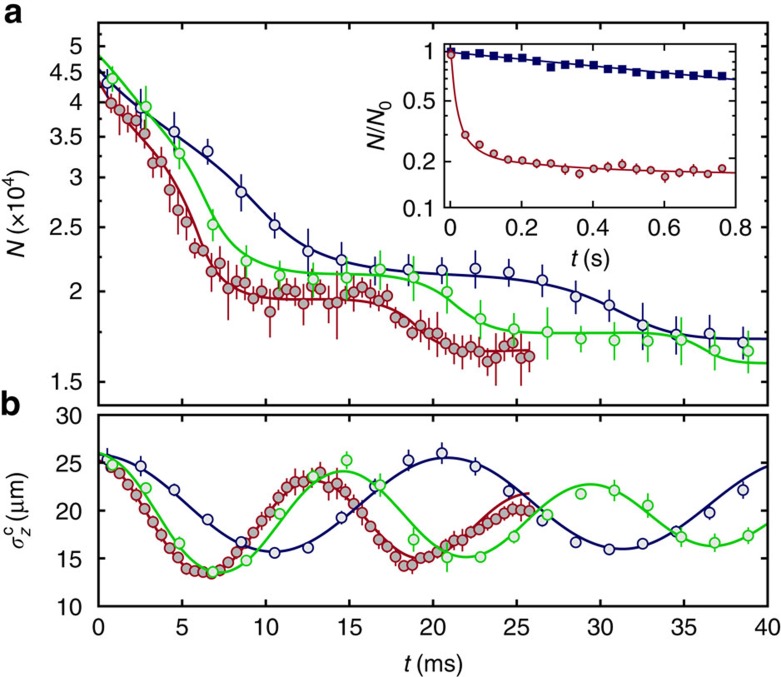
Dynamics of Feshbach molecules for various confinements. (**a**) Decay of the molecule number *N*(*t*) in an ensemble of quasi-1D traps with longitudinal trap frequencies *ω*_*z*_=2*π* × (23.6, 33.4, 49.0) Hz, and transverse frequencies *ω*_*r*_=2*π* × (8.0, 11.6, 17.2) kHz (blue, green, red). The continuous lines are fitted model calculations. The inset shows the typical long-time behaviour of the decay in the quasi-1D traps (red circles) together with the slow decay of immobile molecules in a deep 3D lattice (*ω*_*z*,*r*_=2*π* × 17.2 kHz) (blue squares). *N*_0_ denotes the number of molecules at *t*=0 and the continuous lines are guides to the eye. (**b**) Measured longitudinal width 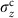 of the molecular cloud. The continuous lines are damped cosines. All data points in **a**,**b** are averages over ∼20 measurements and the error bars indicate the standard mean error.

**Figure 3:**
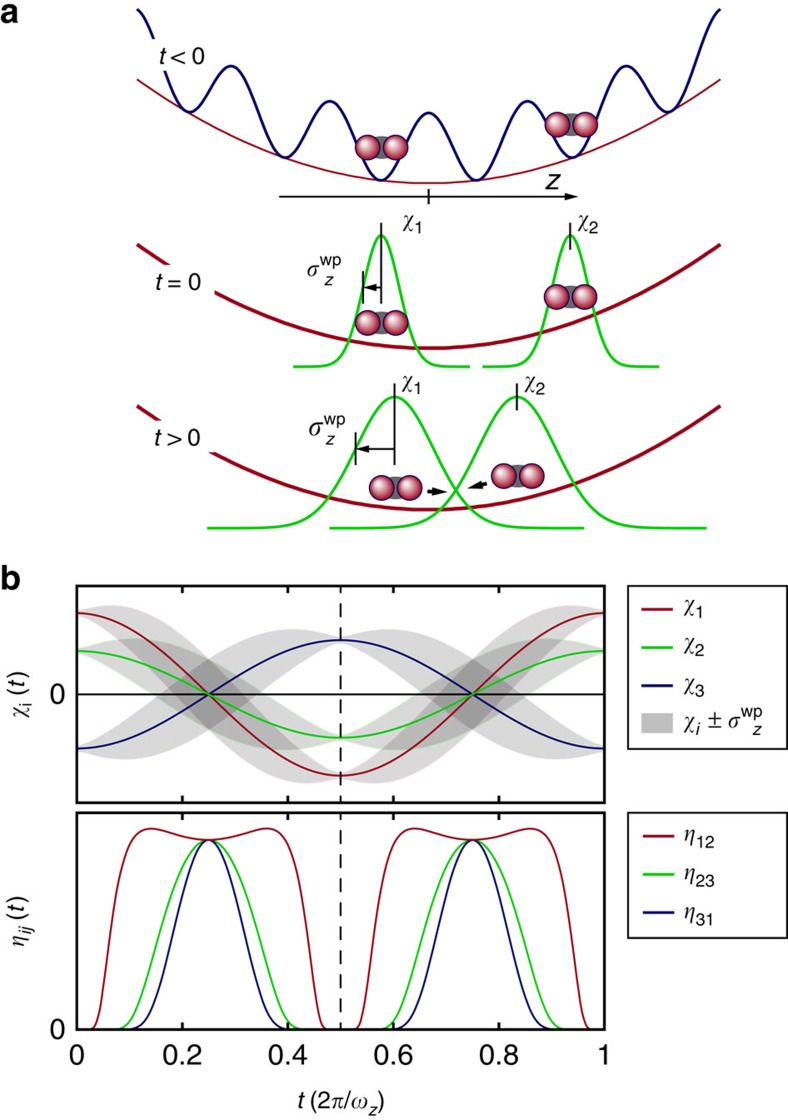
Dynamics of molecular wave packets in a quasi-1D potential tube. (**a**) During the preparation (*t*<0) the molecules are separated by the deep 3D lattice, which suppresses any interaction. At *t*=0 they are released into the quasi-1D tube as Gaussian wave packets. Their centre positions *χ*_*i*_ as well as their width 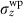 oscillate with *ω*_*z*_ and 2*ω*_*z*_, respectively. (**b**) For the example of three molecules in a tube we show the dynamics of *χ*_*i*_(*t*), 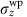
(*t*) (shaded areas) and of the pairwise overlap *η*_*ij*_(*t*).

We now discuss the dynamics within a single 1D tube in more detail. Starting with a classical treatment, we consider each molecule as a point-like particle, which is initially localised and at rest in a single lattice site of the 3D lattice. The molecules are all released exactly at the same time and consequently meet in the centres of the tubes precisely at *t*=*T*/4=*π*/(2*ω*_*z*_). At that particular moment the molecular cloud size ideally vanishes along the *z*-direction, inelastic collisions take place and the total molecule number decreases abruptly. In our measurements, however, we do not observe such abrupt steps, but rather smoothed ones (Fig. 2a). We explain this fact by a non-vanishing initial velocity distribution of the molecules as a result of the Heisenberg uncertainty relation. Therefore, we leave the classical picture of point-like particles and rather describe each molecule *i* as a 1D quantum mechanical wave packet 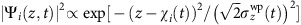, which is centred at *χ*_*i*_(*t*) and has the width 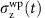 (cf. Fig. 3a). This leads to a finite width in the molecule's velocity distribution, initially given by Δ*v*(0)≈*ħ*/(*m*
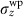
(0)), where *m* is the molecular mass. As *t* progresses, *χ*_*i*_(*t*), 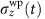 and Δ*v*(*t*) oscillate with *ω*_*z*_, 2*ω*_*z*_ and 2*ω*_*z*_, respectively (Supplementary Note 2). The dynamics for three particles in a tube is depicted in Fig. 3b (upper part). As expected, the collision times are now somewhat smeared out, however, every particle will still pass by every other one in the 1D tube within *T*/2 (assuming that no collisions occur in the meantime). Whenever two molecules collide, there is a certain likelihood for an inelastic process. A collision between two molecules *i* and *j* is possible as long as the spatial overlap 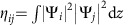 of their wave packets (cf. Fig. 3b, lower part) does not vanish. To describe the loss rate in a single 1D tube we choose the following ansatz (see Supplementary Note 3 for a derivation)


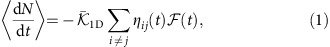


where we sum twice over all molecular pairs (*i*, *j*). 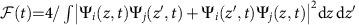 is a normalization factor. 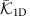 is the mean decay coefficient. We note that in the limit of high tube occupation numbers 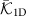 describes the dynamics of a 1D thermal gas with density *n* via the rate equation 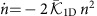.

We use equation (1) to analyse the measured decay curves and to extract 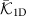. For this, we fit the solution of equation (1) (see Methods) to the measurements. Indeed, the only free fitting parameter in our model is 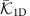. This is because we can experimentally determine the initial spatial distribution of the molecules, and the initial width 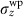
(*t*=0) of the molecular wave packets is derived by calculating the release dynamics from the 3D optical lattice into the 1D tubes (see Supplementary Note 2 and associated Supplementary Fig. 2). For a given molecular sample we assume 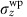
(*t*=0) to be identical for all particles. The values of 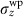
(*t*=0) for the present work range between 0.17 to 0.26*a*_lat_, where *a*_lat_=532 nm is the lattice constant. Generally, the width 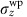
(*t*=0) determines how smoothed out the decay steps are, while 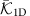 determines their relative heights. Therefore, a variation in 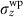
(*t*=0) does not have a strong influence on the extracted rate coefficient 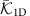. As can be seen in Fig. 2a the fitted curves agree well with the measurements. Our results for the decay rates are 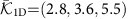 mm s^−1^ for the corresponding radial trap frequencies of *ω*_*r*_=2*π* × (8.2, 11.6, 17.0) kHz, respectively.

We now get back to Fig. 2b. A strong damping of the oscillations of the cloud size 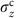
(*t*) along the tubes is observed, which at first might be unexpected for 1D systems where thermalization is generally suppressed^42^. We attribute the damping mainly to the fact that the reaction rate increases with collision energy (as will be shown below) and therefore particles with higher kinetic energy are lost faster. A further discussion also of other possible contributions to the damping is provided in Supplementary Note 4. In addition, we would like to note that the observed oscillating cloud size in Fig. 2b is generally larger than expected from our model calculations. This is due to limitations in the effective imaging resolution (see Methods).

### Collisions of vibrational ground state molecules

**Figure 4:**
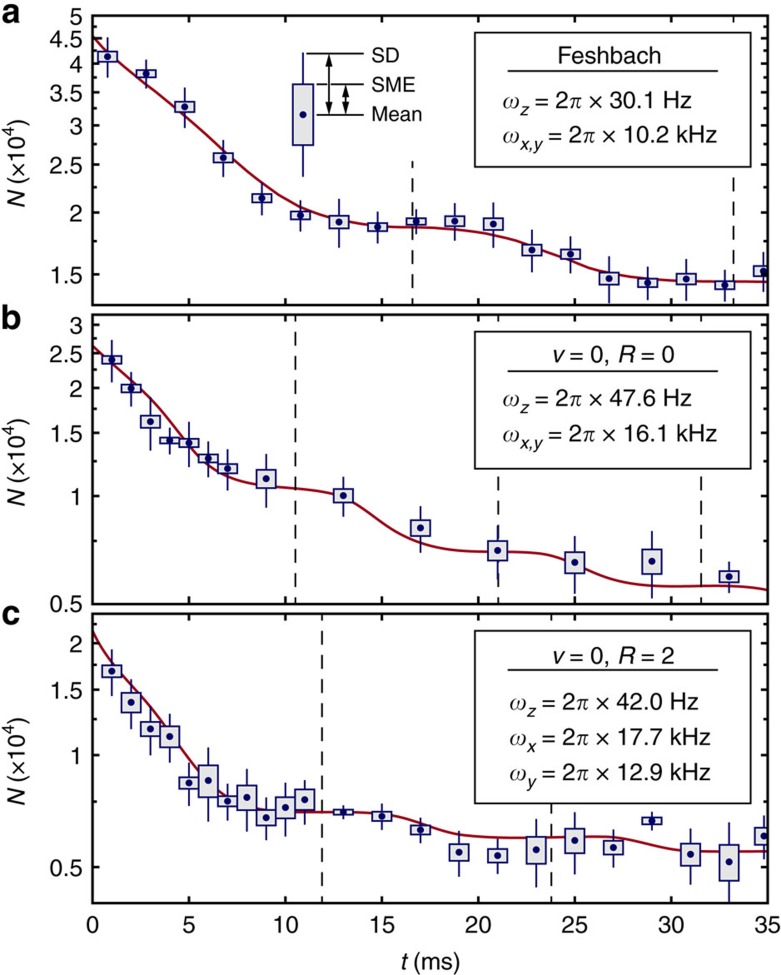
Decay curves for various molecular quantum states. (**a**) Feshbach, (**b**) (*v*=0, *R*=0) and (**c**) (*v*=0, *R*=2) molecules. The laser beam intensities of the 2D lattice are the same within 5% for **a**–**c**, resulting in the trap frequencies provided in the insets. Each data point consists of 10–30 single measurements. In addition to the mean value we give the standard deviation (SD), as well as the standard mean error (SME), represented by the thin and thick bars, respectively (see illustration in **a**). The continuous curves are fitted model calculations based on equation (1). The dashed vertical lines mark multiples of the time *π*/*ω*_*z*_, indicating when the cloud size 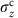 is maximal.

Next, we study the inelastic collisions of the Rb_2_ triplet *v*=0 molecules that are produced in precisely defined internal quantum states via coherent optical transfer starting from the Feshbach state (see Methods). Figure 4b,c shows decay curves of these dimers with rotational quantum number *R*=0 and *R*=2, respectively. As for the Feshbach molecules, the observed loss is almost entirely due to collisions since the measured lifetime in the absence of molecular encounters is on the order of several seconds^43^. For direct comparison, we also present in Fig. 4a a data set obtained with Feshbach molecules. Within 5% the laser intensities of the optical lattice are the same for all three data sets. Remarkably, the measurements clearly reveal that the decay of molecules in state *v*=*R*=0 takes place on a similar timescale as compared to the *v*=0, *R*=2 molecules or the highly excited Feshbach molecules. This is not obvious because the relaxation paths are potentially different for these states. Specifically, while the Feshbach molecules can vibrationally relax within the triplet potential 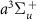, our *v*=*R*=0 molecules are already energetically in the absolute lowest level of the triplet manifold, also with respect to the hyperfine and Zeeman structure (see Methods). Thus, in an inelastic or reactive collision of two of our *v*=*R*=0 molecules either a Rb trimer must form or at least one of the two dimers must undergo a spin flip towards the singlet electronic ground state. Nevertheless, judging from our measurements presented here, there is no indication for a suppression of the molecular loss rate due to these restrictions, which is an important result of our experiments. These findings go along with theoretical predictions for collisions of polar triplet molecules where a spin flip of the electronic state was not suppressed either^44^.

The step-like loss discussed earlier for the Feshbach molecules is also visible in the data on the deeply bound states, albeit less pronounced. The softening of the steps is caused by smaller initial wave packet widths 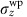
(*t*=0) of the *v*=0 molecules since their polarizabilities at a wavelength of *λ*=1,064 nm are by factors between two and three higher as compared to the Feshbach molecules^45^. This leads to a stronger lattice confinement for the same laser intensities and, in addition, to an earlier non-adiabatic release of the wave packets when the lattice is ramped down.

### Tuning of reaction rates

**Figure 5:**
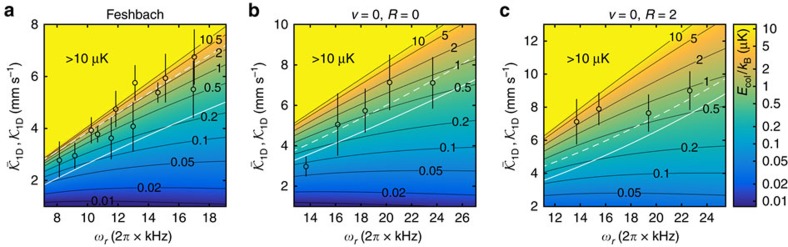
Reaction rate coefficients 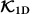 and 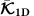. (**a**) Feshbach, (**b**) (*v*=0, *R*=0) and (**c**) (*v*=0, *R*=2) molecules. We show 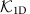 for the universal case, calculated using equation (2). 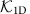 is a function of both *ω*_*r*_ and the collision energy *E*_col_, that is, 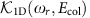. *E*_col_ is displayed in terms of the background colouring and the equi-energy lines (black solid lines) which are given in units of μK × *k*_B_. The black circles represent the values of 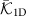 extracted from the experimental decay curves (as in Fig. 4). We note that 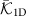 is presented here only as a function of the transverse confinement *ω*_*r*_. As error bars we give the 95% confidence interval of the fits. The solid white lines are the calculations for 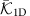 according to equation (3) assuming universality in the reaction model. The white dashed lines correspond to calculations for a non-universal reaction model where the reaction probabilities *P*_re_ are adjusted to describe best the measured data (that is, the black circles). Here the parameter sets for the white dashed lines are (*P*_re_≈0.9, *s*≈15) for the Feshbach molecules, (*P*_re_≈0.9, *s*≈3) for the *v*=0, *R*=0 molecules and (*P*_re_≈0.8, *s*≈−2) for the *v*=0, *R*=2 molecules.

We now investigate the dependence of the reaction rate coefficients on the confinement and collision energy. For this purpose, we measure decay curves for various trap frequencies *ω*_*r*_ of the tubes. Generally, each of these trap frequencies corresponds to a different collision energy *E*_col_, because in our current setup we cannot tune *ω*_*r*_ and *E*_col_ independently as both are controlled via the laser intensity *I* of the optical lattice. Specifically, *ω*_*r*_∝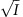 and the collision energy scales as *E*_col_∝
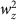
∝*I* due to the initial potential energies of the particles. The decay rate constants 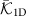 are shown as black circles in Fig. 5 where we use the average trap frequency 
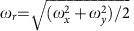
 as a scale for radial confinement (Note that the *v*=0, *R*=2 state exhibits different polarizabilities in *x*- and *y*-direction^45^).

We gain additional insights about the dependencies of the rate coefficient from theoretical considerations. For this, we use a quantum defect reaction model where an inelastic process takes place at short range with probability *P*_re_ (refs 39, 40). On the basis of the experimental observation that the majority of the molecules is lost in their first encounter with another molecule, quite independently of the initially prepared molecular state, we expect the reaction probability *P*_re_ to be close to unity. In the limit that *P*_re_ is unity, we are in the universal regime and the resulting *s*-wave scattering length can be written in a simple form 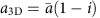 (see Methods for the general formula with *P*_re_≠1). Here 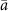
=2*πR*_6_/Γ^2^(1/4) is the mean scattering length of the van der Waals potential^46^, Γ is the gamma function and *R*_6_=(2*μC*_6_*ħ*^−2^)^1/4^ where *μ* is the reduced mass of the molecules. The *C*_6_ coefficient is *C*_6_≈17,550 a.u. (*C*_6_≈18,800 a.u.) for the *v*=0 molecules (Feshbach molecules), respectively^47^. In free space these parameter values would correspond to universal reaction rate constants^39^ 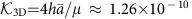 cm^3^ s^−1^ and 1.35 × 10^−10^ cm^3^ s^−1^, respectively, which roughly agree with measured reaction rate constants for ^87^Rb_2_ Feshbach molecules^15^,^24^.

The description changes when reducing the dimensionality of the scattering process^35^,^48^. Generally, a system enters the quasi-1D regime for large trap aspect ratios 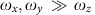 and low enough collision energies *E*_col_<2*ħω*_*x*,*y*_. For our experiments, we estimate the average maximal energies to be a factor of two to three below this boundary (see Supplementary Note 4 and associated Supplementary Fig. 3) and *ω*_*x*,*y*_/*ω*_*z*_ is at least >300 for the three investigated molecular states. Thus, the system can be described by an effective 1D model characterized by the interaction potential 
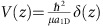
 with complex 1D scattering length *a*_1D_ (refs 49, 50), which is a function of 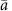 and the trap confinement (see Methods). Parametrizing *a*_1D_ as *a*_1D_=(*α*_1D_−*iβ*_1D_)^−1^ allows for writing the universal 1D reaction rate constant in the form^35^


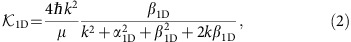


where *ħk* is the relative momentum of the colliding molecules. The rate constant 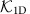 can be considered a function of the collision energy *E*_col_, since *E*_col_=*ħ*^2^*k*^2^/(2*μ*). In addition, through the connection between 1D and 3D scattering lengths, 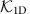 also depends on the transverse confinement *ω*_*r*_, that is, *ω*_*x*_ and *ω*_*y*_ (see equation (6) in Methods). In the low-energy limit, *k*→0, the 1D rate constant 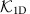 vanishes as *k*^2^, in contrast to 3D where rate constants generally approach a constant value^48^. This is a manifestation of the change of Wigner threshold laws under confinement. To compare scaling of the 1D and 3D cases in more detail we examine the decay rates 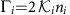, where *i*∈{1D, 3D}, and *n*_1D_ represents a 1D density of molecules. In the low-energy limit, the ratio Γ_1D_/Γ_3D_ is proportional to (*kd*)^2^ × *d*^2^/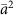 × (*n*_1D_/*d*^2^)/*n*_3D_ (refs 35, 48), where *d* is the transverse confinement size, as given by the harmonic oscillator length (see Methods). We note that *n*_1D_/*d*^2^ can be thought of as an equivalent 3D density of the confined system. The decay rate ratio strongly depends on both confinement and collision energy and indicates that at low enough temperatures and strong confinement the 1D gas would be much more stable than the 3D one.

Figure 5 displays the full dependencies of the universal 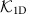 on collision energy and confinement as described by equation (2) for both the Feshbach and the *v*=0 molecules. We note, however, that the calculated values of 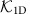 cannot directly be compared to the experimentally determined values of 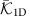 for two reasons. First, the measured 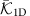 are only given as a function of confinement *ω*_*r*_ and the corresponding average collision energies are not constant. Second, we have to take into account the dynamics of the molecules and their oscillating energy distribution within the lattice. Therefore, we calculate an approximate theoretical 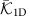 by time-averaging over 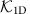 according to


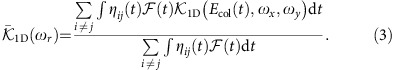


An alternative approach for comparison to the experimental data is described in Supplementary Note 5 (see also Supplementary Fig. 4). In equation (3), the summations cover all possible colliding pairs of molecules (*i*, *j*) in the whole sample with their respective collision energy *E*_col_. The theoretical calculations for 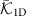 assuming the universal reaction are shown in Fig. 5 as white solid lines. The universal model reproduces the overall slopes of the data, but generally underestimates the measured decay rate constants. This can be explained by a slight non-universal character of the inelastic collisions for all three investigated states as will be shown in the following.

To account for non-universality in our calculations, we use the full expression of *a*_3D_ as given by equation (5) of the Methods section. The theoretical values of 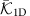 are calculated, again using equations (2) and (3). The non-universal model introduces two free fit parameters, the short-range parameter *s* and the reaction probability *P*_re_ (see Methods). The white dashed lines in Fig. 5 show the results of fits of this non-universal model to the data. For the Feshbach and the *v*=0, *R*=0 molecules good agreement with the measurements is achieved for a wide range of parameter values of *s* at reaction probabilities *P*_re_ between 0.4 and 0.9. The enhancement of the reaction rate relative to the universal regime is due to a shape resonance caused by a near-threshold bound state. For the *v*=0, *R*=2 state the best agreement with the data is obtained when maximizing the calculated rate which is achieved for *P*_re_≈0.8 and a negative *s*. However, still some discrepancy remains. These rotationally excited molecules feature a partial spatial alignment of the molecular axes^45^. Specifically, they collide with their axes pointing dominantly perpendicular to the longitudinal direction of the tubes, which is in contrast to the *R*=0 molecules where the axis distribution is isotropic. This leads to additional quadrupole-quadrupole interactions. We checked, however, that these interactions are far too weak to significantly influence the reaction rates.

## Discussion

In conclusion, we presented the experimental determination of reaction rates for deeply bound triplet molecular states. In general, we observe that the majority of the molecules are already lost in the first encounter. We also demonstrate that the decay rate constant in 1D depends on the confinement strength and the relative energy of the molecules. This offers the possibility to tune the inelastic interaction by adjusting the trap parameters. In this context, it could also be insightful to extend the collision experiments to the quasi-2D and 3D regimes since theory predicts that the dimensionality influences the energy dependence of the reaction rates^35^,^36^,^37^,^38^. Our measurements indicate that inelastic collisions of Rb_2_ molecules are characterized by rate constants close to universality. It would be interesting to pursue the investigation further, by mapping out how the rate constant depends individually on the confinement strength and the relative energy of the molecules. Furthermore, stereochemical aspects can be investigated by adjusting the relative alignment of the molecular axes via the preparation of specific rotational states^45^. The presented experimental results together with the provided theoretical model have to be considered as a step that might pave the way for a fundamental understanding of ultracold chemical reactions and their spatiotemporal control.

## Methods

### Preparation of cold molecules

The molecules are created in an optical lattice which consists of three perpendicular, retro-reflected laser beams of wavelength *λ*=1,064 nm. For this, a thermal sample of roughly 10^6 87^Rb atoms at a temperature of ∼1 μK is loaded into this lattice such that a significant number of sites is doubly occupied. The atoms reside in the lowest Bloch band of the 3D optical lattice and the optical potential is deep enough such that tunnelling is strongly suppressed. To first approximation the atoms are normally distributed in configuration space ∝ 
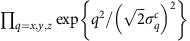
 with the widths of 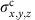
(*t*=0)≈(26, 27, 26) μm. The atomic occupation of each individual lattice site is described by Poisson statistics. The atoms are in the electronic ground state with the total angular momentum quantum numbers *f*=1 and *m*_*f*_=1. By using magnetic Feshbach association at 1007.4 G we create weakly bound *s*-wave molecules at lattice sites occupied with exactly two atoms^41^,^51^. All remaining atoms are removed in a subsequent purification step^41^, such that a pure molecular sample is obtained. The molecular cloud has a size of 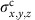
(*t*=0)≈(22, 24, 23) μm after production and contains ∼4.5 × 10^4^ Feshbach molecules. The uncertainties for the given values are ∼10%. Afterwards, for a large part of our experiments, the molecules are transferred from the Feshbach state to the vibrational ground state *v*=0 of the energetically lowest triplet potential 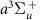. This is done via stimulated Raman adiabatic passage (STIRAP)^1^,^45^ with a transfer efficiency of ∼80%. We prepare the molecules either in one of two quantum levels: The first one is described by the quantum numbers *R*=0, *I*=3, *F*=2, *m*_*F*_=2 where *I*, *F*, *m*_*F*_ denote the total nuclear spin, the total angular momentum and its projection, respectively. This level is energetically the absolute lowest level of the triplet state, see^52^. The second level is rotationally excited by two units of angular momentum and has the quantum numbers *R*=2, *I*=3, *F*=4, *m*_*F*_=4. Compared to the *R*=0 level it has ∼2 GHz × *h* higher energy, see^52^. The clouds of the *v*=0 molecules (both *R*=0 and *R*=2) are smaller than the cloud of Feshbach molecules, that is, 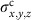
(*t*=0)≈(19, 20, 18) μm, and their particle numbers range from 2.5 × 10^4^ to 3.2 × 10^4^. For the given values the uncertainties are again ∼10%.

### Measuring the molecule number and cloud size

To measure the total number *N* and the cloud size 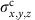 of the molecules in the 1D tubes at a particular point in time the 3D lattice is quickly switched on. This locks the molecules in their current positions. If they are in a *v*=0 state we transfer them back to the Feshbach state using STIRAP and subsequently dissociate them by magnetically ramping back over the Feshbach resonance. The resulting atoms are suddenly released from the optical lattice and after a short (200 μs) time of flight the atom cloud is imaged using standard absorption imaging for another 200 μs. We note that in our imaging procedure the number of molecules is in general underestimated and the measured cloud size is too large. In Supplementary Note 6 (see also Supplementary Fig. 5) we discuss the underestimation of the particle number in detail and derive a correction, which is applied to all our measurements. The overestimated cloud size is a result of the limited resolution of ∼4 μm of the imaging optics and of the expansion of the atomic cloud during time of flight, and during absorption as well as due to tunneling in the 3D optical lattice shortly before detection. This tunneling is enhanced because a sizeable fraction of molecules will be excited to higher Bloch bands when they are rapidly reloaded back into the 3D lattice. This leads to an overestimation of the molecular cloud size 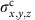 of ∼3 μm. Therefore the relative influence is largest on smaller clouds. While we present the originally measured values for 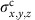 in the text (see also Fig. 2b), we used corrected values for the simulations.

### Determination of trap frequencies

Both, the radial (*ω*_*x*,*y*_) and longitudinal (*ω*_*z*_) trap frequencies are determined from measurements with Feshbach molecules. We use modulation spectroscopy^43^ to obtain *ω*_*x*_ and *ω*_*y*_, respectively, while *ω*_*z*_ is inferred from the periodicity of the steps in the molecular decay curves (cf. Fig. 2). The corresponding trap frequencies for the deeply bound *v*=0 states are derived by comparing their known polarizabilities to the ones of the Feshbach molecules^43^,^45^. As a consistency check, we find agreement between the so-predicted and the experimentally observed periodicity of the decay steps for both *v*=0 states (Fig. 4).

### Numerical integration of the rate equation

To numerically integrate equation (1) we use the random number generated distribution of molecules (Fig. 1b), which assigns an initial location *χ*_*i*_(*t*=0) to each particle *i*. Next, we propagate the wave packets of all molecules in small time steps Δ*t*. The decay probability of each molecule pair (*i*, *j*) during Δ*t* is given by 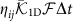. If an inelastic collision takes place, both involved particles are removed from the sample.

### Scattering in quasi-1D geometry

Since in our setup 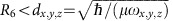, the collision in the presence of the trap can be described within the pseudopotential approximation with (in general) energy-dependent 3D scattering length^50^,^53^. In this treatment the interaction potential is replaced by the regularized Dirac delta function and in addition an effective 1D model can be derived^49^, using 
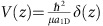
. The resulting 1D scattering length *a*_1D_ is connected to the 3D one via^54^,


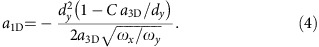


Here *C* is a numerical factor depending only on the transverse trap anisotropy *ω*_*x*_/*ω*_*y*_. For the *R*=0 state (and the Feshbach state) *C*=−*ζ*(1/2)≈1.46, where *ζ* denotes the Riemann zeta function, while for the *R*=2 state *C*≈1.57. The complex valued 3D scattering length can be written as^40^,


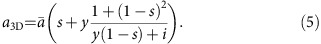


Here *y* is defined via *P*_re_=4*y*/(1+*y*)^2^, where *P*_re_ denotes the short-range inelastic process probability. The parameter *s* is the value of the scattering length in the absence of inelastic collisions in units of 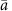.

The short-range reaction probability *P*_re_ approaches unity in the limit *y*→1. Inserting *y*=1 into equation (5), one observes that the scattering length approaches *a*_3D_=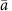
(1−*i*) regardless of the value of *s*. This means that the reaction dynamics becomes universal in the sense that it is independent of the short-range details of the potential, which normally determine the scattering length. For *y*=1, equation (4) can be rewritten as


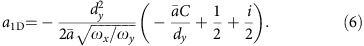


### Data availability

The data that support the findings of this study are available from Björn Drews on reasonable request.

## Additional information

**How to cite this article:** Drews, B. *et al*. Inelastic collisions of ultracold triplet Rb_2_ molecules in the rovibrational ground state. *Nat. Commun.* **8,** 14854 doi: 10.1038/ncomms14854 (2017).

**Publisher's note**: Springer Nature remains neutral with regard to jurisdictional claims in published maps and institutional affiliations.

## Supplementary Material

Supplementary Information Supplementary Figures, Supplementary Notes and Supplementary References.
