# Supplementary material for: Inelastic collisions of ultracold triplet Rb2 molecules in the rovibrational ground state
Source: Nat Commun. 2017 Mar 23;8:14854. doi: 10.1038/ncomms14854 (PMC5376650; doi:10.1038/ncomms14854)
Supplement: Supplementary Information — Supplementary Figures, Supplementary Notes and Supplementary References. [file ncomms14854-s1.pdf]

**Supplementary Note 1: Effects of the STIRAP transfer**

The STIRAP transfer of the Feshbach molecules to the $v = 0$ state is not perfect. The overall transfer efficiency is typically about 80%. This produces a fraction of molecules in undetected quantum states (i.e. dark molecules) which, however, still contribute to reactive collisions. In addition, the STIRAP transfer is spatially inhomogeneous. This is due to the different dynamical polarizabilities of the Feshbach state and the $v = 0$ states, which in the harmonic dipole trap leads to a spatially varying two-photon resonance frequency. Thus, if the two-photon transition is driven resonantly at the centre of the particle cloud, the energy detuning $\Delta E(x,y,z)$ varies as

$$\Delta E(x,y,z) = \frac{m}{2}(\omega_{v=0}^2 - \omega_{\mathrm{FB}}^2)(x^2 + y^2 + z^2), \tag{1}$$

where $m$ is the molecular mass. $\omega_{\mathrm{FB}} = 2\pi \times 44\,\mathrm{Hz}$ is the trap frequency of the Feshbach molecules, while $\omega_{v=0} = 2\pi \times 70\,\mathrm{Hz}$ is the trap frequency for the $v = 0, R = 0$ state. The blue (continuous) curve in Supplementary Fig. 1a shows $\Delta E(x,y,z)$ along the $z$-axis, i.e. $\Delta E(x = 0, y = 0, z)$. In parallel measurements we have investigated how the STIRAP efficiency depends on the relative detuning of the Raman beams. From these investigations we find that the transfer efficiency can be well described by

$$P(x,y,z) = 0.85 \cdot \exp\left(\frac{-(\Delta E/\hbar)^2}{2\xi^2}\right), \tag{2}$$

where $\xi \approx 2\pi \times 100\,\mathrm{kHz}$. The factor of 0.85, which represents the maximum efficiency obtained at the centre of the trap, is adjusted to give an overall cloud transfer probability of 80%. Supplementary Fig. 1a shows $P(x = 0, y = 0, z)$ (red dashed line). Its width can be compared to the extension of the initial sample of Feshbach molecules which is illustrated by the shaded distribution.

The imperfect and spatially inhomogeneous STIRAP transfer has a direct influence on the decay signals of the molecules. In Supplementary Fig. 1b we compare our measurements with the model predictions (which take into account dark molecules and their inelastic collisions). For this purpose, we consider the number of remaining observable molecules $N_\infty$ at long times, i.e. after all collisions have taken place. These molecules are trapped in singly occupied tubes. $N_0$ is the total initial number of observable molecules at $t = 0$, i.e. when they are released into the 1D tubes. The data points are directly extracted from the measurements, after subtracting the slow exponential decay due to loss mechanisms other than cold collisions. The dashed (solid) lines are the results of our calculations of the molecular decay for the Feshbach molecules ($v = 0$ molecules). Indeed, the agreement between the measurements and the calculations for the Feshbach state is good, supporting our model for the molecular distribution of Feshbach molecules over the tubes. Now we consider the $v = 0$ states. For a perfectly efficient STIRAP transfer, the data points would fall on the dashed line (which is not the case). If we include the finite transfer efficiency (see Supplementary Eq. (2)) of our STIRAP we obtain the solid line which is in agreement with the data points.

Similar agreement is found for the $v = 0, R = 2$ state. The reduction in the ratio $N_\infty/N_0$ can partially be explained as follows. As can be seen from Supplementary Fig. 1a, the inhomogeneous STIRAP transfer is less efficient in the outer parts of the molecular cloud where it dominantly consists of molecules in singly occupied tubes. These account for the main contribution to the $N_\infty$ signal. An imperfect STIRAP transfer reduces $N_\infty$ and thereby $N_\infty/N_0$.

**Supplementary Note 2: Molecular wave packets - size and dynamics**

In our model we assume that initially each molecule in the 3D lattice is localised in a single lattice site where it is found in the energetically lowest trap state. It's wave function is then approximately described by a Gaussian wave packet. The dynamics of this Gaussian wave packet after release from the 3D lattice into the 1D tube can be described by the analytical solutions[1] for the wave packet centre $\chi(t)$, the wave packet size $\sigma_z^{\mathrm{wp}}(t)$ and the momentum width $\sigma_p(t)$:

$$\chi(t) = \chi(0)\cos(\omega_z t), \tag{3}$$

$$\sigma_z^{\mathrm{wp}}(t) = \sigma_z^{\mathrm{wp}}(0)\left[\cos^2(\omega_z t) + \left(\frac{\hbar \sin(\omega_z t)}{2m(\sigma_z^{\mathrm{wp}}(0))^2\omega_z}\right)^2\right]^{\frac{1}{2}}, \tag{4}$$

$$\sigma_p(t) = \frac{\hbar}{2\sigma_z^{\mathrm{wp}}(0)}\left[\cos^2(\omega_z t) + \left(\frac{2m\omega_z(\sigma_z^{\mathrm{wp}}(0))^2\sin(\omega_z t)}{\hbar}\right)^2\right]^{\frac{1}{2}}, \tag{5}$$

where $\omega_z$ is the longitudinal trap frequency of the 1D tubes. $\sigma_z^{\mathrm{wp}}(0)$ is the initial width and $\chi(0)$ is the initial centre position of the wave packet at $t = 0$, i.e. directly after the release. In the following, we focus on $\sigma_z^{\mathrm{wp}}(t)$ as it is relevant for the description of the molecular decay in our model. Since the initial width $\sigma_z^{\mathrm{wp}}(0)$ cannot be measured directly, we estimate it by numerically calculating how the wave packet in the 3D lattice changes during the ramp-down of the optical potential $U_z$ in the longitudinal direction. This ramp lowers the potential from $U_z^0$ to zero within 400 µs (see inset of Supplementary Fig. 2a). The lattice potentials in the $x$- and $y$-directions are kept constant at $U_x^0$ and $U_y^0$, respectively. They determine the trap frequencies $\omega_{x,y,z}$ for the collision experiments in the 1D tubes. In Supplementary Fig. 2a the red curve displays how the wave packet size $\sigma_z^{\mathrm{wp}}$ at $t = 0$ depends on the initial potential depth $U_z^0$. $U_z^0$ is given in units of the recoil energy $\mathrm{E_r} = h^2/(2m\lambda^2)$, where $m$ is the mass of a molecule and $\lambda$ is the wavelength of the lattice laser, i.e. $\lambda = 1064\,\mathrm{nm}$. A smaller value of $U_z^0$ leads to a larger width $\sigma_z^{\mathrm{wp}}(t=0)$. This is because a decreasing $U_z^0$ leads to a slower ramp speed for which, in turn, the molecular wave packet can follow the changing lattice adiabatically for a longer time. Thus, the wave packet is released from the lattice with a larger size $\sigma_z^{\mathrm{wp}}(t=0)$. Our calculations show that for the ramps used in the experiments the wave packet still very much resembles a Gaussian at $t = 0$. This is also revealed when we plot $\hbar/(2\sigma_p(t = 0\,\mu\mathrm{s}))$ (see green line in Supplementary Fig. 2a), which should be identical to $\sigma_z^{\mathrm{wp}}(t=0)$ for a Gaussian wave packet. Indeed, for $U_z^0 > 20\,\mathrm{E_r}$ both curves are quite close. For comparison we also show the wave packet size before the ramp-down at $t = -400\,\mu\mathrm{s}$ (blue curve).

In our model calculations of the molecular decay we use the calculated values for $\sigma_z^{\mathrm{wp}}(t=0)$ given in Supplementary Fig. 2a. The ranges of potential depths $U_z^0$ for our measurements on the Feshbach and the $v = 0$ molecules to extract the reaction rate coefficients are indicated by the grey horizontal bars. For Feshbach molecules this corresponds to a range of $\sigma_z^{\mathrm{wp}}(t=0) = 0.20 - 0.26\,a_{\mathrm{lat}}$ while for the $v = 0$ states

the values are within $\sigma_z^{\mathrm{wp}}(t=0) = 0.17 - 0.21\, a_{\mathrm{lat}}$.

In Supplementary Fig. 2b we present model calculations of molecular decay curves for a range of $\sigma_z^{\mathrm{wp}}(t=0)$ between 0.09 and $0.30\, a_{\mathrm{lat}}$. This range is somewhat larger than our experimental one, such that we can display the general dependence of the decay curve on $\sigma_z^{\mathrm{wp}}(t=0)$. Initial small wave packets lead to smoothing out of the decay steps while larger initial wave packets lead to a more pronounced step-like behaviour. However, the relative heights of the steps, which mainly determine $\overline{\mathcal{K}}_{\mathrm{1D}}$, do not change considerably. Therefore, a moderate variation in $\sigma_z^{\mathrm{wp}}(t=0)$ has no strong effect on the extracted decay coefficient $\overline{\mathcal{K}}_{\mathrm{1D}}$.

We can qualitatively confirm this behaviour in our experiments. Supplementary Fig. 2c shows three measurements for the potential depths $U_z^0 = [14, 32, 51]\, \mathrm{E_r}$. According to Supplementary Fig. 2a these depths correspond to wave packet widths $\sigma_z^{\mathrm{wp}}(t=0) = [0.28, 0.23, 0.21]\, a_{\mathrm{lat}}$, respectively.

**Supplementary Note 3: Derivation of rate equation**

In the following we derive the rate equation (1) of the main part of the paper. Let $\Psi_i(z,t)$ denote the (normalized) wave function of particle $i$ (in our case $\Psi_i(z,t)$ is a Gaussian wave packet). Then, the wave function of two bosonic particles $i$, $j$ is

$$\Psi_{ij}(z,z',t) = \frac{1}{\sqrt{\mathcal{N}(t)}} \left(\Psi_i(z,t)\Psi_j(z',t) + \Psi_i(z',t)\Psi_j(z,t)\right), \tag{6}$$

where $\mathcal{N}(t) = \int dz dz' \left|\Psi_i(z,t)\Psi_j(z',t) + \Psi_i(z',t)\Psi_j(z,t)\right|^2$ is the normalization factor which ensures that the two-particle wave function is normalized for each $t$, i.e. $\int dz dz' |\Psi_{ij}(z,z',t)|^2 = 1$. Because we are only considering contact interactions here, the probability $d^2P_\mathrm{R}(i,j)$ for a reaction to take place within the infinitesimal intervals $dz$ and $dt$ has to be proportional to the probability of finding both particles within $dz$:

$$\begin{aligned} d^2P_\mathrm{R}(i,j) &= \mathcal{K}_\mathrm{1D}|\Psi_{ij}(z,z'=z,t)|^2 \, dz dt \\ &= \mathcal{K}_\mathrm{1D}|\Psi_i(z,t)|^2|\Psi_j(z,t)|^2\mathcal{F}(t)\, dz dt, \end{aligned} \tag{7}$$

where $\mathcal{F}(t) = \frac{4}{\mathcal{N}(t)}$ and $\mathcal{K}_\mathrm{1D}$ is a rate constant which in general depends on the details of the collision, such as the collision energy. Strictly speaking, the collision energy is not precisely defined here, because of the momentum uncertainty within the wave packets. Thus, $\mathcal{K}_\mathrm{1D}$ is effectively a mean value of the rate constant.

If more than two particles are present, the total probability for a two-body reaction $d^2P_\mathrm{R}$ is obtained by summing over all particle pairs,

$$\begin{aligned} 2\, d^2P_\mathrm{R} &= \sum_{i\neq j} d^2P_\mathrm{R}(i,j) \\ &= dz dt \sum_{i\neq j} \mathcal{K}_\mathrm{1D} \, |\Psi_i(z,t)|^2|\Psi_j(z,t)|^2\mathcal{F}(t). \end{aligned} \tag{8}$$

Here, the factor of two comes about due to double counting of the pairs. Next, we make an approximation by simplifying Supplementary Eq. (8) to read

$$2\, d^2P_\mathrm{R} = \overline{\mathcal{K}}_\mathrm{1D}\, dz dt \sum_{i\neq j} |\Psi_i(z,t)|^2|\Psi_j(z,t)|^2\mathcal{F}(t), \tag{9}$$

where $\mathcal{K}_\mathrm{1D}$ is replaced by an average rate constant $\overline{\mathcal{K}}_\mathrm{1D}$ which depends on the mean collision energy of the whole ensemble. For our analysis this approximation is quite convenient but not fully justified since in our experiment the collision energy distribution varies as a function of time and space. Still we decided to work with an average rate constant $\overline{\mathcal{K}}_\mathrm{1D}$, because this reduces the number of fit parameters to a minimum and thus assures that the fits to the data are meaningful. $2d^2P_\mathrm{R}$ corresponds to the expectation value for the particle loss within $dz$ and $dt$,

$$-\langle d^2N\rangle = d^2P_\mathrm{R} \times 2 + \left[1 - d^2P_\mathrm{R}\right] \times 0 = 2\, d^2P_\mathrm{R}, \tag{10}$$

where we take into account that two particles are lost in each reaction. Integrating over space yields

$$\langle dN \rangle = -\overline{\mathcal{K}}_{1\mathrm{D}}\, dt \sum_{i\neq j} \int |\Psi_i(z,t)|^2 |\Psi_j(z,t)|^2 dz\, \mathcal{F}(t), \tag{11}$$

which is identical to Eq. (1) of the main part of the paper.

Next we show that Supplementary Eq. (11) is equivalent to the conventional two-body decay equation for a Bose-Einstein condensate. For this, we make use of Supplementary Eqs. (9) and (10) and we assume that all particles have the same density distribution $|\Psi_i(z,t)|^2 = n_i = n_1$ and thus $\mathcal{F}(t)$=1. Furthermore, the total particle number $N = \sum_i 1$ is large. Then, one obtains

$$\begin{aligned} \langle d^2N \rangle &= -\overline{\mathcal{K}}_{1\mathrm{D}}\, dz\, dt \sum_{i\neq j} n_1^2 \\ &= -\overline{\mathcal{K}}_{1\mathrm{D}}\, dz\, dt\, N(N-1) n_1^2 \\ &\approx -\overline{\mathcal{K}}_{1\mathrm{D}}\, dz\, dt\, N^2 n_1^2 \\ &= -\overline{\mathcal{K}}_{1\mathrm{D}}\, dz\, dt\, n^2, \end{aligned} \tag{12}$$

where $n = N n_1$ is the density distribution of the gas. Since $\langle d^2N/(dzdt) \rangle = dn/dt$, it follows

$$\dot{n} = -\overline{\mathcal{K}}_{1\mathrm{D}} n^2, \tag{13}$$

which is the well known rate equation. In addition, we have numerically confirmed that Supplementary Eqs. (11) and (13) yield the same evolution for wave packets propagating in a box potential.

We point out that in a many-body system the decay process may be affected by the correlations present in the gas and its kinetic properties. For example, in the work of Stoof *et al.*[2] reactive losses from a BEC and a thermal gas are discussed. Since the $g^{(2)}$ correlation function of a thermal gas is by a factor of two larger as compared to the one of a BEC (in the limit of vanishing interparticle distances), the reaction rate coefficient is also by a factor of two higher. Specifically, the inelastic loss for a thermal ensemble of molecules can be characterized by $\dot{n} = -\overline{\mathcal{K}}^{\mathrm{th}}_{1\mathrm{D}} n^2$, similar to Supplementary Eq. (13), however, the corresponding rate coefficient is given by $\overline{\mathcal{K}}^{\mathrm{th}}_{1\mathrm{D}} = 2 \times \overline{\mathcal{K}}_{1\mathrm{D}}$. Please note that in the present paper all results are given in terms of $\overline{\mathcal{K}}_{1\mathrm{D}}$. In this context we would like to mention the work of Dürr *et al.*[3] for the description of a dissipative Tonks-Girardeau gas, where the correlations strongly suppress the decay.

**Supplementary Note 4: Damping of cloud oscillations and distribution of collision energies**

In Fig. 2b of the main text we observe a damping in the oscillations of the Feshbach molecule cloud size $\sigma_z^c$. Damping should not occur in a true 1D system where only elastic two-body collisions are relevant. Therefore we explain the damping mainly as a consequence of inelastic two-body collisions. As the reaction rate increases with the relative collision energy $E_{\mathrm{col}}$, highly energetic molecules are lost faster, which effectively corresponds to damping. Our simulations indeed show, that between 5% and 10% of the amplitude is lost during the first oscillation period. In order to verify the dependence of the damping on the collision energy, we carry out experiments where we measure the damping for various confinements $\omega_z$, since $E_{\mathrm{col}}$ scales with $\omega_z^2$. For each $\omega_z$, the damping constant $\gamma$ is extracted by fitting a function $f(t) = ae^{-\gamma t}\cos(\omega_z t) + b$ to the data. Supplementary Fig. 3a shows the results for the measurements on Feshbach molecules and reveals a linear dependence of $\gamma/\omega_z$ on the longitudinal trap frequency $\omega_z$, i.e. $\gamma \propto \omega_z^2$. Since $1/\omega_z$ sets the overall time scale for collisions in our setup, the quadratic dependence confirms our explanation.

Finally, we also check that we are well enough in the 1D regime such that elastic two-body collisions do not significantly contribute to the damping. If we were not deeply in the 1D regime, an elastic collision of two molecules could convert part of the collision energy $E_{\mathrm{col}}$ into radial excitation energy $2\hbar\omega_r$ towards the first transverse lattice band. For symmetry reasons such a process requires both particles to be excited to the first band. The transferred portion of energy is missing in the longitudinal motion and thus the oscillation amplitude along this direction is reduced. Assuming a Gaussian distribution of molecules and a known longitudinal trap frequency $\omega_z$, we calculate the distribution of collision energies. For this, we consider for each pair of molecules $i$ and $j$ in a tube its maximal relative energy $E_{\mathrm{col}}^{\mathrm{max}}$. Supplementary Fig. 3b shows a corresponding probability distribution of $E_{\mathrm{col}}^{\mathrm{max}}$ for a typical sample of Feshbach molecules (see figure for cloud parameters).

Noticeably, the fraction of events where the collision energy exceeds $2\hbar\omega_r$ is relatively low. We estimate this by counting the possible molecule collisions with a maximal relative energy of $E_{\mathrm{col}}^{\mathrm{max}} > 2\hbar\omega_r$. According to Supplementary Fig. 3c the percentage is below 3% for all measurements on Feshbach molecules. Together with the fact that most of the collisions are inelastic, we conclude that non-1D effects in the description of the collisions are negligible.

**Supplementary Note 5: Direct calculations of molecular decay curves based on universal model**

The rate coefficient given by Eq. (2) of the main part of the publication can be applied to directly calculate the expected molecular decay curves, when we take into account the time-dependence of the oscillating distributions of the molecules in both configuration and momentum space. For this, rate equation (1) of the main part of the publication has to be modified to read

$$\left\langle \frac{dN}{dt} \right\rangle = -\sum_{i\neq j} \mathcal{K}_{1\mathrm{D}}(E_{\mathrm{col}}(t), \omega_x, \omega_y)\eta_{ij}(t)\mathcal{F}(t). \tag{14}$$

As a consequence of assuming universality, there is no free adjustable parameter. Supplementary Fig. 4 shows these full calculations (dashed lines) along with the data and fit curves of Fig. 4 of the main part of the publication. For the calculations we use the same values for $\sigma_z^{\mathrm{wp}}$, $\sigma_{x,y,z}^{\mathrm{c}}$ and $N(t=0)$ as for the fit curves. The overall agreement between the data and the universal model calculations is quite reasonable considering the rigidity of the model and the numerous experimental parameters with their respective uncertainties.

**Supplementary Note 6: Imperfect particle number measurement**

As mentioned in the main text, our detection method underestimates atom and molecule numbers at low densities. In the following analysis, we derive a density dependent correction factor which is applied to all measurements in order to obtain the real number of molecules. We first discuss an exemplary measurement: an atomic sample in a harmonic trap is abruptly released for a variable time of flight before it is detected via absorption imaging. During time of flight the cloud expands and its density decreases, while the total number of particles $N$ is conserved. However, as can be seen from Supplementary Fig. 5a the total number $\widetilde{N}$ obtained from absorption imaging in our setup decreases as a function of the peak (= central) 2D density $d_{\mathrm{max}}$ during expansion. We note that in order to better compare Supplementary Fig. 5a to the other plots the atom number and density is given in 'molecular units', i.e. $2 \times \widetilde{N}$ is the atom number and $2 \times d_{\mathrm{max}}$ is the atomic density.

The data of Supplementary Fig. 5a can be used directly to correct the shortcomings of the detection process down to peak molecular densities of about 1 molecule $\times$ $\mu$m$^{-2}$. In order to obtain a correction factor for even lower densities, we use a refined method where we compare the measured density distribution of a cloud of molecules to its known shape. Generally, when a thermal cloud is released from a harmonic trap the expanding density distribution is Gaussian in all three spatial dimensions. Imaging the cloud integrates along one direction and yields a 2D Gaussian distribution. Our measured distributions deviate from a 2D Gaussian. To show this, we first fit a Gaussian function

$$d_{\mathrm{g}}(y,z) = d_{\mathrm{max}} \exp\left( -\frac{(y-\mu_y)^2}{2(\sigma_y^{\mathrm{c}})^2} - \frac{(z-\mu_z)^2}{2(\sigma_z^{\mathrm{c}})^2} \right) \tag{15}$$

to an absorption image (see inset of Supplementary Fig. 5b). Here, $(\mu_y, \mu_z)$ is the centre position of the distribution and $(\sigma_y^{\mathrm{c}}, \sigma_z^{\mathrm{c}})$ are the widths of the function along the respective axis. For fitting, only the dense part of the particle distribution is considered (with more than 1.2 molecules $\times$ $\mu$m$^{-2}$). In order to reduce the noise level on our data we carry out azimuthal averaging. For this, we first convert the measured 2D distribution to a circular symmetric 2D distribution by mapping each image pixel with its polar coordinates $(r,\phi)$ to the rescaled coordinates $(r' = r/\sigma(\phi), \phi)$ where the width $\sigma(\phi)$ is determined by $1/\sigma^2(\phi) = \cos^2(\phi)/(\sigma_y^{\mathrm{c}})^2 + \sin^2(\phi)/(\sigma_z^{\mathrm{c}})^2$. Afterwards, we bin the data in the $r'$-direction and average over $\phi$ to obtain the radial density distribution $d_{\mathrm{m}}$ as shown in Supplementary Fig. 5b. If the values of $d_{\mathrm{m}}$ are compared to the radial density distribution $d_{\mathrm{g}}$ of the Gaussian fit, systematic deviations for densities below 1 molecule $\times$ $\mu$m$^{-2}$ are revealed. For each $r' = r/\sigma(\phi)$, we now calculate the ratio between $d_{\mathrm{m}}$ and $d_{\mathrm{g}}$ and average it over more than one hundred images which are obtained for the same initial experimental parameters. These mean values are shown as circles in Supplementary Fig. 5c while the red curve is an interpolation $f(d_{\mathrm{g}})$ which reaches essentially unity for densities above 1.8 molecules $\times$ $\mu$m$^{-2}$. In order not to overestimate the signal loss effect, we conservatively set $f(0) = 0.5$. $f(d_{\mathrm{g}})$ is a transfer function which can be used to

calculate the signal loss for the total number of molecules (as observed in Supplementary Fig. 5a) and to generate a density dependent correction factor. The Gaussian distribution $d_{\rm g}(y,z)$ of Supplementary Eq. (15) is multiplied with the transfer function $f(d_{\rm g}(y,z))$. Integration yields $\widetilde{N} = \int f(d_{\rm g}(y,z))d_{\rm g}(y,z)dydz$, while $N = \int d_{\rm g}(y,z)dydz$. Based on this we can determine the ratio $\widetilde{N}/N$ as a function of the observed peak density $d_{\rm max}$ (see continuous line in the inset of Supplementary Fig. 5c). As a consistency check we also show the data from Supplementary Fig. 5a, with $N \approx 5\times10^4$ molecules and find good agreement.

Now we are able to correct for the underestimated particle numbers in the measurements. For a given central cloud density $d_{\rm max}$, the measured numbers of molecules are multiplied by the inverse of $\widetilde{N}/N$. Since the Feshbach samples in our experiments exhibit $d_{\rm max} > 2.5$ molecules $\times$ $\mu$m$^{-2}$, the correction factor is comparably low here. In contrast to that, typical central densities for the $v=0$ molecules are between 1 and 0.1 molecules $\times$ $\mu$m$^{-2}$ which leads to a relative correction factor of about 1.4 between high and low particle numbers in a set of data. One example is given in the left part of Supplementary Fig. 5d. It shows the raw data of a molecular decay curve for $v=0, R=0$ together with the corrected particle numbers. The step-like structure is partially distorted by the correction process and the heights of the steps become slightly smaller. In Supplementary Fig. 5d (right) we compare how the decay rate constant $\overline{\mathcal{K}}_{\rm 1D}$ changes after correcting the particle numbers. This is done for all our measurements on $v=0, R=0$ molecules. Clearly, the change is not very significant and typically lies within the error margins.

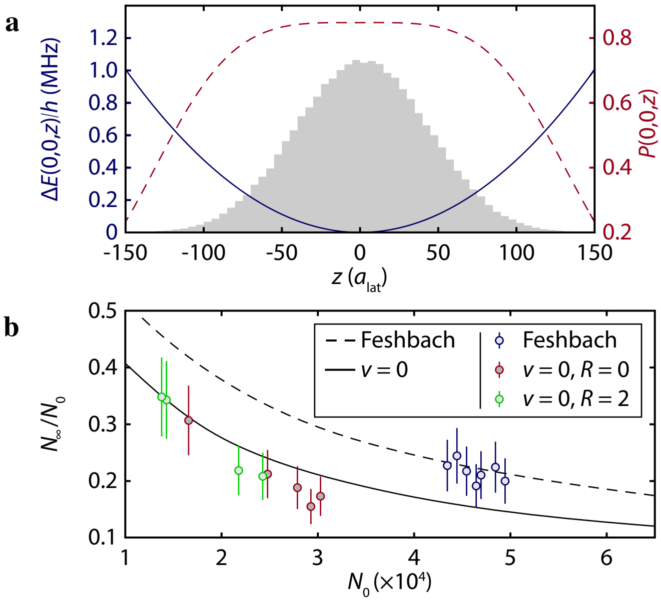


**Supplementary Figure 1. Imperfect STIRAP transfer and its consequences. a**, The blue solid line shows the frequency shift $\Delta E(0,0,z)/h$ of the two-photon STIRAP resonance as a function of the position. Here, $a_{\mathrm{lat}}$ is the lattice constant. The frequency shift causes a spatially inhomogeneous transfer probability $P(0,0,z)$ (red dashed line). For comparison, the shaded area depicts the approximate distribution of Feshbach molecules in arbitrary units. **b**, The ratio of initial ($N_0$) and final ($N_\infty$) molecule numbers is plotted as a function of $N_0$, for both Feshbach and $v = 0$ molecules. The plot symbols show measurements, dashed and continuous lines are corresponding calculations as discussed in the text. The error bars are calculated using the combined systematic and statistical uncertainties of $\pm 5\%$ and $\pm 10\%$ in the values of $N_0$ and $N_\infty$, respectively, which are extracted from the experimental data.

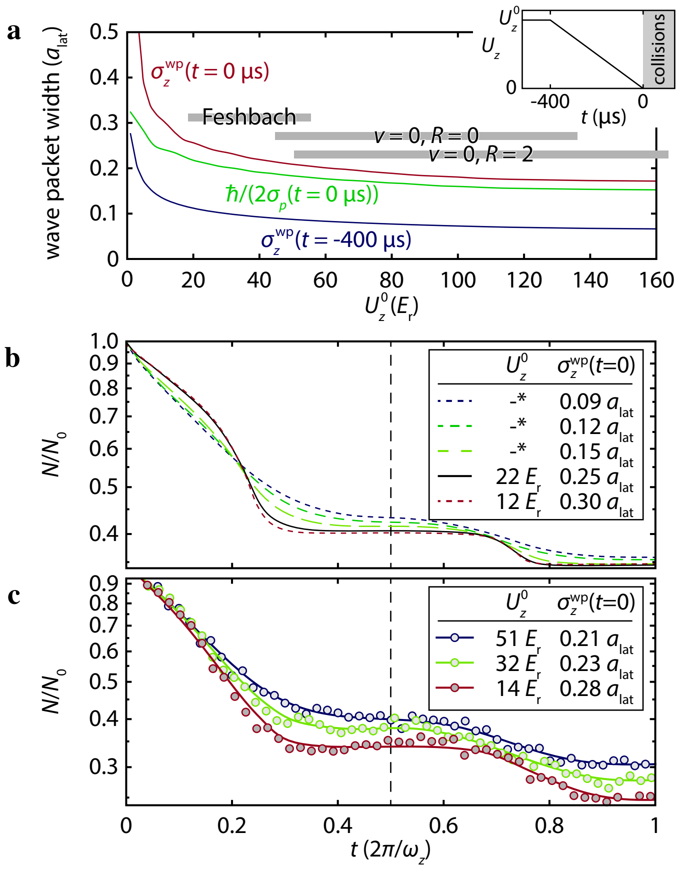


**Supplementary Figure 2. Wave packet size and influence on molecular decay dynamics. a**, Numerical simulation of molecular wave packet widths. The plot shows $\sigma_z^{\mathrm{wp}}(t = 0\,\mu\mathrm{s})$ (red) and $\hbar/(2\sigma_p(t = 0\,\mu\mathrm{s}))$ (green) of a released molecule after linearly ramping down the optical lattice in the longitudinal direction from the initial depth $U_z^0$ at $t = -400\,\mu\mathrm{s}$ to zero at $t = 0\,\mu\mathrm{s}$, where the collision experiment starts (see inset). For comparison, we also present the spatial width $\sigma_z^{\mathrm{wp}}(t = -400\,\mu\mathrm{s})$ (blue) of the trapped particle in the initial 3D optical lattice. The grey horizontal bars give the range of lattice depths $U_z^0$ for all our collision experiments on the three individual molecular states, which were used to extract the reaction rate coefficients. **b**, Comparison of calculated molecular decay curves for different initial widths $\sigma_z^{\mathrm{wp}}(t = 0) = [0.09, \ldots, 0.30]\, a_{\mathrm{lat}}$ of the molecular wave packets after release into the 1D tubes. The corresponding values of $U_z^0$ are provided in the inset, where the star symbols mark potential depths that cannot be reached in our setup. For the calculations we used the parameters $N_0 = 4.5 \times 10^4$, $\overline{\mathcal{K}}_{\mathrm{1D}} = 120\ a_{\mathrm{lat}}/\omega_z$ and $\sigma^{\mathrm{c}}_{x,y,z}(t = 0) = (20, 22, 22)\ \mu\mathrm{m}$. The dashed vertical line indicates the time of a half oscillation of the molecular cloud. **c**, Measured decay curves of Feshbach molecules. They differ in the ramping speeds (see inset) when releasing the particles into the 1D tubes. The continuous lines are guides to the eye.

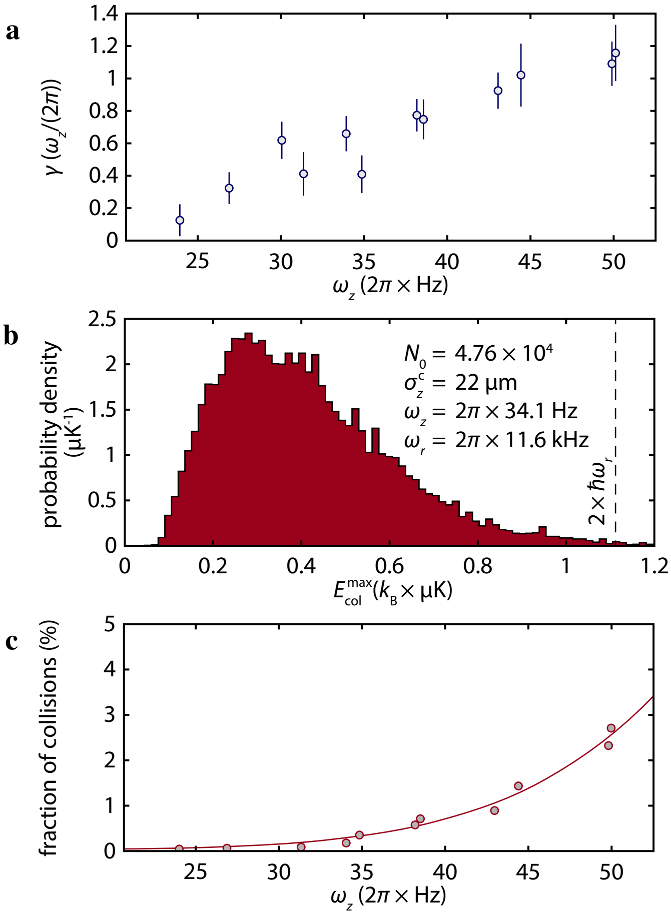


**Supplementary Figure 3. Damping constant and energy distribution. a**, Damping constant $\gamma$ of the oscillations in the width $\sigma_z^c$ of the molecular cloud, extracted from measurements on Feshbach dimers (cf. Fig. 2b of the main text). The error bars represent the 95% confidence intervals of the fits. We note that $\gamma$ is given in units of the inverse oscillation period, so it represents the damping per oscillation. **b**, Normalized histogram of the maximal relative collision energies $E_{\rm col}^{\rm max}$ for a sample of Feshbach dimers with the parameters specified in the inset. The dashed vertical line marks the value of $E_{\rm col}^{\rm max} = 2\hbar\omega_r$. **c**, Fraction of colliding molecule pairs with $E_{\rm col}^{\rm max}$ larger than $2\hbar\omega_r$. The circles are obtained from reconstructed energy distributions using the extracted parameters from the measurements of **a**. The solid line is a guide to the eye.

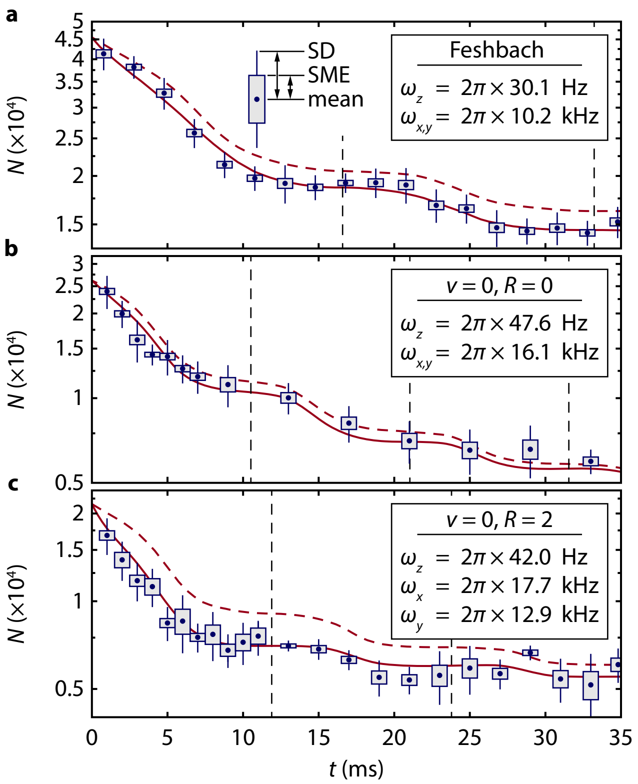


**Supplementary Figure 4. Comparison of model predictions to the data for various molecular quantum states.** **a**, Feshbach, **b**, $(v = 0, R = 0)$ and **c**, $(v = 0, R = 2)$ molecules. We show the plot of Fig. 4 of the main part of the publication together with the dashed curves, which are calculations based on Supplementary Eq. (14). For the experimental data the standard deviation (SD), as well as the standard mean error (SME) are given by the thin and thick bars, respectively (see illustration in **a**).

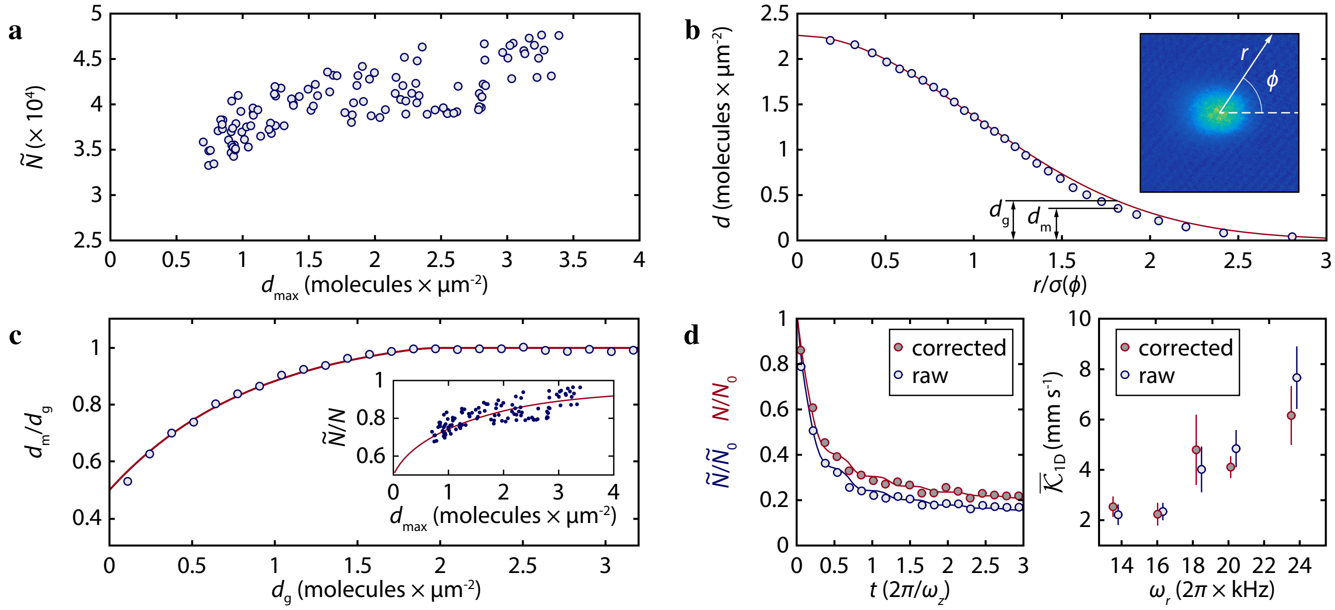


**Supplementary Figure 5. Signal loss in the imaging process.** **a**, Apparent loss in the observed total particle number $\widetilde{N}$ as a function of the density $d_{\mathrm{max}}$ at the centre of the expanding atom cloud. **b**, Deviation of the measured molecular cloud from a Gaussian population distribution. The locally determined density of molecules $d_{\mathrm{m}}$ (circles) is shown versus the (rescaled) radial distance $r/\sigma(\phi)$. It is compared to a Gaussian distribution $d_{\mathrm{g}}$ (solid line), which is fitted to the dense central region of the cloud. The plot represents the azimuthal average (see text) of the image in the inset. **c**, Ratio of the local densities $d_{\mathrm{m}}/d_{\mathrm{g}}$ dependent on $d_{\mathrm{g}}$ with the solid line being the interpolation $f(d_{\mathrm{g}})$ of the data points. In the inset, we plot the measurements of (**a**) (circles) in terms of the relative particle number $\widetilde{N}/N$, where $N$ denotes the real number of particles expected from our reconstructed density distribution (solid line). **d**, (left) shows a measured decay curve of an ensemble of $v=0$, $R=0$ molecules with trap frequencies of $\omega_z = 2\pi\times 40.2\,\mathrm{Hz}$ and $\omega_r = 2\pi\times 13.7\,\mathrm{kHz}$, either with (red) or without (blue) correction for the signal loss, where the solid lines are the corresponding simulations. The molecule numbers are normalized to their respective initial value at $t=0$. The plot on the right-hand side summarizes the resulting values of $\overline{\mathcal{K}}_{1\mathrm{D}}$ for all data on $v=0$, $R=0$ molecules, i.e. different radial trap frequencies $\omega_r$, both for corrected and raw particle numbers. For better visibility the circles are slightly shifted with respect to each other in the horizontal direction. As error bars we give the 95% confidence interval of the fits.

## Supplementary References


[1] Sakurai, J. J. *Modern Quantum Mechanics* Rev edn (Addison-Wesley, 1995).

[2] Stoof, H. T. C., Janssen, A. M. L., Koelman, J. M. V. A. & Verhaar, B. J. Decay of spin-polarized atomic hydrogen in the presence of a Bose condensate. *Phys. Rev. A* **39,** 3157 (1989).

[3] Dürr, S. *et al.* Lieb-Liniger model of a dissipation-induced Tonks-Girardeau gas. *Phys. Rev. A* **79,** 023614 (2009).
